# The Data Efficiency of Deep Learning Is Degraded by Unnecessary Input Dimensions

**DOI:** 10.3389/fncom.2022.760085

**Published:** 2022-01-31

**Authors:** Vanessa D'Amario, Sanjana Srivastava, Tomotake Sasaki, Xavier Boix

**Affiliations:** ^1^Department of Brain and Cognitive Sciences, Massachusetts Institute of Technology, Cambridge, MA, United States; ^2^Center for Brains, Minds and Machines, Cambridge, MA, United States; ^3^Department of Computer Science, Stanford University, Stanford, CA, United States; ^4^Artificial Intelligence Laboratory, Fujitsu Limited, Kawasaki, Japan

**Keywords:** data efficiency, overparameterization, object recognition, object background, unnecessary input dimensions, deep learning

## Abstract

Biological learning systems are outstanding in their ability to learn from limited training data compared to the most successful learning machines, *i.e.*, Deep Neural Networks (DNNs). What are the key aspects that underlie this data efficiency gap is an unresolved question at the core of biological and artificial intelligence. We hypothesize that one important aspect is that biological systems rely on mechanisms such as foveations in order to reduce unnecessary input dimensions for the task at hand, *e.g.*, background in object recognition, while state-of-the-art DNNs do not. Datasets to train DNNs often contain such unnecessary input dimensions, and these lead to more trainable parameters. Yet, it is not clear whether this affects the DNNs' data efficiency because DNNs are robust to increasing the number of parameters in the hidden layers, and it is uncertain whether this holds true for the input layer. In this paper, we investigate the impact of unnecessary input dimensions on the DNNs data efficiency, namely, the amount of examples needed to achieve certain generalization performance. Our results show that unnecessary input dimensions that are task-unrelated substantially degrade data efficiency. This highlights the need for mechanisms that remove task-unrelated dimensions, such as foveation for image classification, in order to enable data efficiency gains.

## 1. Introduction

The success of Deep Neural Networks (DNNs) contrasts with the still distant goal of learning with few training examples as in biological systems, *i.e.*, in a data efficient manner (Hassabis et al., 2017). Understanding the principles that underlie such differential is a question at the core of both artificial and biological intelligence. In this paper, we introduce the hypothesis that an important aspect for data efficiency is that biological systems rely on mechanisms such as foveations in order to reduce unnecessary input dimensions, *e.g.*, background in object recognition, while state-of-the-art DNNs do not.

DNNs are usually trained on high dimensional datasets (*e.g.*, images and text), and many input dimensions of the DNN may be unnecessary to predict the ground-truth label as they are unrelated and/or redundant to the task at hand. Machine learning theory for linear and kernel methods predicts that unnecessary input dimensions may degrade the DNN's data efficiency (Hastie et al., 2009), as the classifier may overfit to the unnecessary input dimensions if not enough training examples are provided to learn to discard them.

However, DNNs have challenged classic machine learning measures of complexity (*e.g.*, VC dimensions, Rademacher complexity) as they can achieve high test accuracy despite having a number of trainable parameters much larger than the number of training examples, *i.e.*, DNNs are overparameterized (Zhang et al., 2017; Nakkiran et al., 2020). Since unnecessary input dimensions lead to more overparameterization, it is unclear in what way DNNs suffer from unnecessary input dimensions and whether more data is needed to learn to discard them.

To foreshadow the results, we find that the DNNs' data efficiency depends on whether the unnecessary dimensions are *task-unrelated* or *task-related* (redundant with respect to other input dimensions). Namely, increasing the number of *task-unrelated* dimensions leads to a substantial drop of data efficiency, while increasing the number of *task-related* dimensions that are linear combinations of other *task-related* dimensions, helps to alleviate the negative impact of the *task-unrelated* dimensions. These results suggest that mechanisms to discard unnecessary input dimensions, such as foveations for object recognition, are necessary to enable data efficiency gains.

## 2. Related Works

We now relate our work with the effect of background on the generalization abilities of DNNs in object recognition, and also with the DNNs generalization abilities depending on the number of parameters of the network.

### 2.1. Object's Background and DNN Generalization

The data collection process is often biased (Torralba and Efros, 2011). One of the most prominent factors of such dataset bias is the background, such that some aspects of the background systematically co-occur with certain objects, *e.g.*, airplanes may tend to always appear in the sky. This co-occurrence is a confounding factor for the network, and the network may learn to associate the background with the object, *e.g.*, the sky may be regarded as part of the airplane. Previous works have shown that DNNs for image recognition fail to classify objects in novel and uncommon backgrounds (Choi et al., 2012; Volokitin et al., 2017; Beery et al., 2018; Tian et al., 2018). Remarkably, popular object recognition datasets are biased to such an extent that DNNs can predict the object category even when the objects are removed from the image (Zhu et al., 2017; Tian et al., 2018; Xiao et al., 2021). Barbu et al. (2019) introduced a new benchmark which addresses the biased co-occurrence of objects and background, among other types of bias. DNNs exhibit large performance drops in this benchmark compared to ImageNet (Deng et al., 2009). Recently, Borji has shown that a large portion of the performance drop comes from the bias in object's background, as classifying the object in isolation substantially alleviates the performance drop (Borji, 2021).

In contrast to previous works, we analyse the impact of the object's background to the DNN's generalization performance when the dataset is unbiased, *i.e.*, there is no significant correlation between the objects and backgrounds and the statistics of the object's background are the same between training and testing times. To the best of our knowledge, our work is the first to investigate the effects of object's background on DNNs when these are unbiased. We show that just the presence of background, even if it is unbiased, can degrade the data efficiency of the DNN.

### 2.2. Overparameterization and Data Dimensionality

A remarkable characteristic of DNNs is that the test error follows a double-descend when the DNN's width is increased by adding more hidden units. Thus, the test error decreases as the network's width is increased in both the underparameterized and overparameterized regimes, except in a critical region between these two where a substantial error increase can take place (Belkin et al., 2019; Advani et al., 2020; Nakkiran et al., 2020). The overparameterized regime has received a lot of attention because DNNs with many more parameters than training examples can achieve high test accuracy, and a theoretical understanding of this phenomenon is an active area of research. Robustness to overparametrization relates to unnecessary input dimensions because unnecessary input dimensions also increase the number of parameters of the network, albeit in the input layer rather than in the intermediate layers. As we show in the sequel, increasing the number of unnecessary input dimensions can have the opposite effect of increasing the number of hidden units in the test error.

A theoretical understanding of this phenomenon using mathematical tools is an open question. The PAC Bayes theory appears as a promising approach to describe the generalization capacity of DNNs [*e.g.*, (Dziugaite and Roy, 2017; De Palma et al., 2019; Bernstein and Yue, 2021)]. While these theoretical results provide insights about the trends of the behaviour of the DNN, an empirical, quantitative assessment of the effect of unnecessary dimensions to the DNN's data efficiency is missing. Our analysis derives from theoretical insights of the exact solution of a linear network in a regression task. In this way, we can relate and compare empirical results for DNNs with cases that are well understood theoretically.

Another strand of research relates the structure of the dataset with the generalization ability of the network. Several works in statistical learning theory for kernel machines relate the spectrum of the dataset with the generalization performance (Zhang, 2005). For neural networks, Ansuini et al. (2019); Recanatesi et al. (2019) define the intrinsic dimensionality based on the dimension of the data manifold. These works analyze how the network reduces the intrinsic dimension across layers. Yet, these metrics based on manifolds do not provide insights about how specific aspects of the dataset, *e.g.*, unnecessary dimensions, contribute to the intrinsic dimensionality.

## 3. Unnecessary Input Dimensions and Data Efficiency

We aim at analyzing the effect of unnecessary input dimensions on the data efficiency of DNNs. Let **x** be a vector representing a data sample, and let **y** be the ground-truth label of **x**. We define *f*(**x**) = **y** as the target function of the learning problem. Also, we use [**x**; **u**] to denote the data sample **x** with unnecessary input dimensions appended to it. The unnecessary dimensions do not affect the target function of the learning problem, *i.e.*, *g*([**x**; **u**]) = *f*(**x**) = **y**, where *g* is the target function of the learning problem with unnecessary input dimensions. Each sample can have a different set of dimensions that are unnecessary, *e.g.*, one sample could be [**x**_1_; **u**_1_] and another be [**u**_2_; **x**_2_]. Note that this variability is present in object recognition because the dimensions representing the object's background are unnecessary and vary across data samples, as the object can be in different image locations.

We define two types of unnecessary input dimensions: *task-unrelated* and *task-related*. Unnecessary input dimensions are *task-unrelated* when they are independent of **x**, *i.e.*, they can not be predicted from **x**, as in unbiased object's background. Otherwise, the unnecessary dimensions are *task-related*, which are equivalent to redundant dimensions. An example that leads to more *task-related* unnecessary dimensions is upscaling the image.

To study the effect of unnecessary input dimensions, we measure the test accuracy of DNNs trained with different amounts of unnecessary input dimensions and training examples. Given a DNN architecture and a dataset with a fixed amount of unnecessary dimensions, we define the *data efficiency* of the DNN as the Area Under the Test Curve (AUTC) for the DNN trained with different number of training examples. The curve is monotonically increasing, as more training examples lead to higher test accuracy, and the AUTC measures the area under it. We normalize the AUTC to be between 0 and 1, where 1 is the maximum achievable, and it corresponds to 100% test accuracy for all number of training examples. In the experiments where the number of training examples spans several orders of magnitude, we calculate the AUTC by converting the number of training examples in logarithmic scale, such that all orders of magnitude are equally taken into account.

## 4. Datasets and Networks

We now introduce the datasets and networks we use in the experiments (refer to Appendices 1, 2 for additional details).

### 4.1. Linearly Separable Dataset

We use a linearly separable dataset for binary classification, as it facilitates relating results of classic machine learning and DNNs. We generate a binary classification dataset of 30 input dimensions, which follow a Gaussian distribution with (μ = 0, σ = 1). The ground-truth label is the output of a linear classifier, such that the dataset is linearly separable with a hyperplane randomly chosen. Unnecessary input dimensions are appended to the data samples. *Task-unrelated* dimensions follow a Gaussian distribution with (μ = 0, σ = 0.1). The *task-related* dimensions are linear combinations of the dimensions of the original dataset samples.

We evaluate the following linear and Multi-Layer Perceptron (MLP) networks: linear network trained with square loss (pseudo-inverse solution), MLP with linear activation functions trained with either square loss or cross entropy loss, and MLP with ReLU trained with cross entropy loss.

### 4.2. Non-linearly Separable Dataset With Different Noise Distributions

To further evaluate the generality of results on data distributions that are not linearly separable, we use a mixture of Gaussians to generate non-linearly separable datasets for binary classification. Each class consists of three multivariate Gaussians of dimensions *p* = 30. We generate a sample by randomly selecting with the same probability one of the three distribution. To give a more comprehensive evaluation on the effect of different types of noise, we generate unnecessary dimensions using Gaussian distributions with different variance, and we also evaluate two other noise distributions, namely, Gaussian noise with Σ_*ii*_ = 1, ∀*i*, with Σ_*ij*_ = 0.5, ∀*i*≠*j*, and salt and pepper noise, where each vector component can assume value (0, or *u*), based on a Bernoullian distribution on {−1, 1}.

We consider the MLP with ReLU and soft-max with cross-entropy loss because among the different variants it is the only well suited to fit non-linearly separable data.

### 4.3. Object Recognition Datasets

We evaluate object recognition datasets based on extensions of the MNIST dataset (LeCun et al., 1998) and the Stanford Dogs dataset (Khosla et al., 2011).

**Synthetic and Natural MNIST**. We generate two datasets based on MNIST: the synthetic MNIST and the natural MNIST, which have synthetic and natural background, respectively. In both datasets, the MNIST digit is always at the center of the image and normalized between 0 and 1.

In the synthetic MNIST dataset, the *task-unrelated* dimensions are sampled from a Gaussian distribution with (μ = 0, σ = 0.2) and the *task-related* dimensions are the result of upscaling the MNIST digit. We also combine *task-related* and *unrelated* dimensions by fixing the size of the image and changing the ratio of *task-related* and *unrelated* dimensions by upscaling the MNIST digit.

In the natural MNIST dataset, the background is taken from the Places dataset (Zhou et al., 2014), as in Volokitin et al. (2017). The size of the image is constant across experiments (256 × 256 pixels), and the size of the MNIST digits determines the amount of *task-related* and *unrelated* dimensions.

We use the MLP with ReLU and cross entropy loss, and also Convolutional Neural Networks (CNNs). The architecture of the CNN consists of three convolutional layers each with max-pooling, followed by two fully connected layers. Since the receptive field size of the CNN neurons may have an impact on the data efficiency, we evaluate different receptive field sizes. We use a factor *r* to scale the receptive field size, such that the convolution filter size is (*r*·3) × (*r*·3) and the pooling region size is (*r*·2) × (*r*·2). We experiment by either fixing *r* to a constant value or adapting *r* to the scale of the MNIST digit, such that the receptive fields of the neurons capture the same object region independently of the scale of the digit.

**Stanford Dogs**. Recall our analysis focuses on unnecessary input dimensions that are unbiased. We use the Stanford Dogs dataset (Khosla et al., 2011) as it is reasonable to assume that the bias between breeds of dogs and background is negligible. This dataset contains natural images (227 × 227 pixels) of dogs at different image positions. The amount of *task-unrelated* dimensions is determined by the dog size, which is different for each image. To evaluate the effect of unnecessary input dimensions, we introduce the following five versions of the dataset. Case 1 corresponds to the original image. In case 2, we multiply by zero the pixels of the background, which reduces the variability of the *task-unrelated* dimensions. In case 3, the dog is centered in the image. In case 4, we fix the ratio of *task-related/unrelated* dimensions by centering the dog and scaling it to half of the image size. In case 5, we remove the background by cropping and scaling the dog.

We use a ResNet-18 (He et al., 2016), following the standard pre-processing of the image used in ImageNet.

## 5. Results

In this section, we report results, first on the linearly separable datasets and then, on the object recognition datasets.

### 5.1. Linearly Separable Dataset

**Figure 1:**
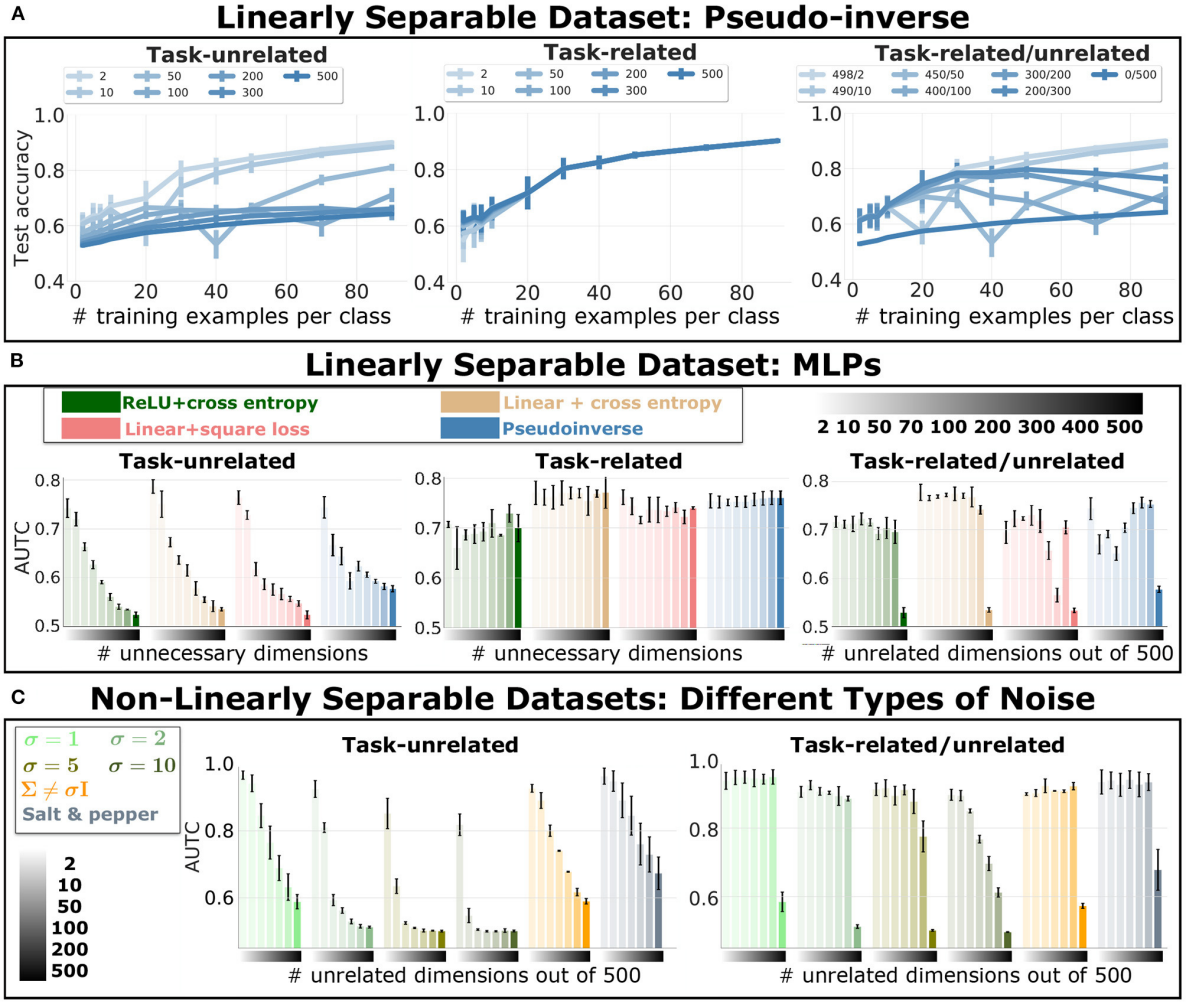
*Data Efficiency of Linear and Fully Connected Networks Trained on Dataset For Binary Classification*. Data efficiency for different amount of unnecessary input dimensions. Error bars indicate standard deviation across experiment repetitions. **(A)** Test accuracy of the pseudo-inverse solution as function of different amount of training examples. Each curve corresponds to a different amount of additional *task-unrelated* (left plot), *task-related* (middle plot) and *task-related/unrelated* dimensions, as reported in the legend. **(B)** We report the Area Under the Test Curve (AUTC) of the accuracy for different amount of training examples. We indicate with the gradient bar on the *x*-axis the amount of additional *task-unrelated* dimensions (left and right plot), and additional *task-related* dimensions, in the middle plot. The legend indicate the different networks trained and tested on the dataset. **(C)** AUTC values for an MLP with ReLU activation and cross entropy loss, for different types of *task-unrelated* dimensions: Gaussian independent components with increasing variance, Gaussian with non diagonal covariance, and salt and pepper noise. The gradient on the *x*-axis indicates the number of *task-unrelated* dimensions.

Figure 1A shows the test accuracy of the pseudo-inverse solution for different number of training examples and unnecessary input dimensions. Figure 1B reports the data efficiency of all networks tested for different number of unnecessary input dimensions. Recall that the data efficiency is measured with the AUTC and summarizes the test accuracy as a function of the amount of training examples, *e.g.*, for the pseudo-inverse solution, the curves in Figure 1A are summarized by the AUTC in Figure 1B. We observe that increasing the amount of *task-unrelated* dimensions harms the data efficiency, *i.e.*, the AUTC drops. Also, the *task-related* dimensions alone do not harm data efficiency, and they alleviate the effect of the *task-unrelated* dimensions.

These results clarify the difference between robustness to overparameterization in intermediate layers and unnecessary input dimensions. Note that the effect on the test accuracy of increasing the number of hidden units is the opposite of increasing the number of *task-unrelated* input dimensions, *i.e.*, DNNs are not robust to all kinds of overparameterization.

Analytical results for linear regression using the square loss predicts an analogous effect of *task-unrelated* dimensions on the solution. For sake of clarity, we retrace these results in the Appendix 3.1, where we outline the effect of additional *task-unrelated* dimensions that are Gaussian-distributed on the pseudo-inverse solution. There, we show that *task-unrelated* dimensions lead to the pseudo-inverse, Tihkonov-regularized solution calculated in the dataset without *task-unrelated* dimensions. Since in this case the regularization can not be tuned or switched off as it is fixed by the number of *task-unrelated* dimensions, it is likely to harm the test accuracy, as we have observed.

The regularizer is beneficial in some specific cases. Following from regularization theory (Hastie et al., 2009), Appendix 3.2 highlights a noisy regression problem in which certain amounts of *task-unrelated* dimensions help to improve generalization. In a classification problem, Tiknhonov regularization may also be beneficial in some cases. This can be seen in Figure 1A, where we observe that for a given number of training examples, increasing the number of *task-unrelated* dimensions improves the test accuracy in some cases. This specific trend relates to the aforementioned double descend of DNNs (Belkin et al., 2019; Advani et al., 2020). As shown in Nakkiran et al. (2020), the location of the critical region is affected by the number of training examples and the complexity of the model. Here, the complexity of the model is affected by the number of *task-unrelated* dimensions due to its regularization effect.

### 5.2. Non-linearly Separable Datasets With Different Distributions of *Task-Unrelated* Dimensions

In Figure 1C, we show the data efficiency of MLP with ReLU trained with cross entropy loss on non-linearly separable datasets. On the left of the quadrant, we report different distributions of the *task-unrelated* dimensions: Gaussian noise with different ***σ*** (corresponding to multiplicative factor applied on the identical covariance matrix), Gaussian noise with non-diagonal covariance matrix and salt and pepper noise. The amount of *task-unrelated* dimensions is reported through the colored bar (indicated with a gradient). We observe that, similarly to previous results in the linearly separable dataset (Figure 1B), *task-unrelated* dimensions harm data efficiency. Also, as expected, data efficiency deteriorates as the variance of Gaussian noise increases. The combination of *task-related/unrelated* dimensions alleviates the detrimental effect of *task-unrelated* dimensions. These empirical results on MLPs show a similar trend to the one predicted for linear networks, with exception of a less pronounced effect of the double descent behavior.

### 5.3. Object Recognition Datasets

**Figure 2:**
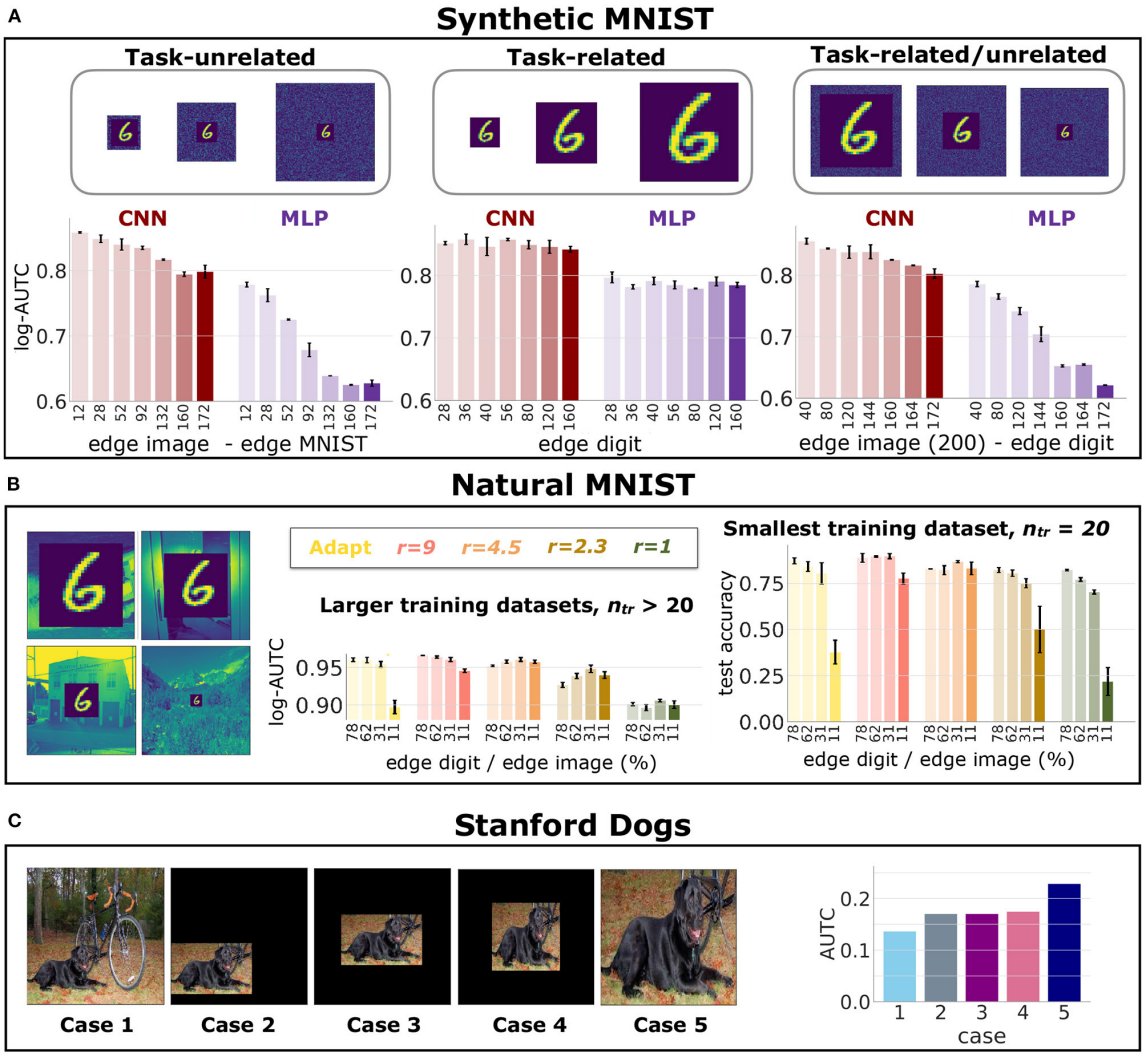
*Data Efficiency in Object Recognition Datasets*. Results of DNNs' data efficiency for different amount of unnecessary input dimensions, tasks and networks. Error bars indicate standard deviation across experiment repetitions. **(A)** log-AUTC for CNNs and MLPs trained on synthetic MNIST, for different number of unnecessary dimensions. **(B)** Left plot: log-AUTC for networks trained on Natural MNIST for larger amount of training examples; Right: test accuracy on the smallest training set. **(C)** AUTC on the Stanford Dogs dataset for the five cases shown on the left of the panel.

Figure 2A shows the log-AUTC for the MLP and the CNN for different amount of unnecessary dimensions (an increasing amount as we move left to right), for the synthetic MNIST dataset. In Appendix 4.1, we report the test accuracy for different number of unnecessary dimensions, which further strengthens the results of Figure 1. Conclusions are consistent with the previous results in the linearly separable dataset. Also, we observe that CNNs are overall much more data efficient than MLPs, which is expected because of their more adequate inductive bias given by the weight sharing of the convolutions.

Figure 2B shows results in natural MNIST dataset for different ratios of *task-related/unrelated* dimensions. The plots compare CNNs with different receptive field sizes, represented by the factor *r* (see Section 4.3). Since the CNN achieves high accuracy with few examples, the mean and standard deviation of the log-AUTC (left plot) hardly show any variation when computed on more than 20 training examples per class. Yet, the gap of the testing accuracy is considerable for 20 training examples per class (right plot). These results confirm that *task-unrelated* dimensions degrade data efficiency independently of the receptive field sizes (see Appendix 4.2 for additional results further supporting these conclusions).

Figure 2C shows results on the Stanford Dogs dataset, namely the AUTC score across the five cases of unnecessary dimensions that we evaluate. This dataset serves to assess a more realistic scenario, where the objects can appear at different positions and scales. We observe that the *task-unrelated* dimensions, which come from the background, harm the data efficiency (cases 1 to 4 versus case 5). Putting to zero the unnecessary dimensions improves the data efficiency of models trained on the original dataset (cases 2 to 4 vs. case 1). This is because the *task-unrelated* dimensions become redundant as they all take the same value in all images. We also observe that removing the variability of the position and scale of the object hardly affects the data efficiency (case 2 to 4). Thus, learning to discard the background requires more training examples than learning to handle the variability in scale and position of the object.

## 6. Conclusions

We have analyzed the effect of unnecessary input dimensions (*e.g.*, object's background). We found that *task-unrelated* dimensions harm the data efficiency, while increasing the number of *task-related* dimensions that are linear combinations of other *task-related* dimensions help to alleviate the negative effect of *task-unrelated* dimensions. These results demonstrate that the robustness of DNNs to overparameterization is limited, as increasing the number of *task-unrelated* input dimensions is a form of overparameterization that degrades the accuracy. Also, our results add to the growing body of works in object recognition that shows that bias in the object's background can undermine the reliability of DNNs. Here we have shown that the problem runs far deeper, as the object's background negatively affects the network even when there is no bias.

Taken together, these results suggest that data efficiency gains could be enabled by mechanisms that remove *task-unrelated* dimensions, such as foveation for image classification (Luo et al., 2016; Akbas and Eckstein, 2017), or also by adapting to DNNs regularization techniques that encourage predictions from a sparse subset of input dimensions (*e.g.*, ℓ_1_ regularization for linear regression Hastie et al., 2019). Also, our results can be extended to other domains, such as natural language processing and clinical tasks, as the effect of unnecessary dimensions may have been investigated, *e.g.*, (Laksana et al., 2020), but their effects in the data efficiency remain largely unexplored.

## Data Availability Statement

Publicly available datasets were analyzed in this study. This data can be found at: Stanford Dogs dataset: https://www.tensorflow.org/datasets/catalog/stanford_dogs; MNIST dataset: https://www.tensorflow.org/datasets/catalog/mnist; PLACES dataset: http://places.csail.mit.edu/; Synthetic datasets can be generated using the following code: https://github.com/vanessadamario/data_efficiency/blob/main/synthetic_framework/main.py; The code supporting the conclusions of this article is publicly accessible in the following github repository: https://github.com/vanessadamario/data_efficiency.git.

## Author Contributions

VD'A implemented the experiments and carried out the analysis, with contributions of SS and XB. VD'A and XB conceived the experiments with contributions of SS and TS. VD'A and XB wrote the manuscript with contributions of TS. XB and TS supervised the study. All authors contributed to the article and approved the submitted version.

## Funding

This work has been supported by the Center for Brains, Minds, and Machines (funded by NSF STC award CCF-1231216), XB by the R01EY020517 grant from the National Eye Institute (NIH) and XB and VD'A by Fujitsu Laboratories Ltd. (Contract No. 40008819) and the MIT-Sensetime Alliance on Artificial Intelligence.

## Conflict of Interest

This study received funding from Fujitsu Laboratories Ltd. The funder through TS had the following involvement with the study: conception of the experiment, writing of this article, and supervision of the study. The remaining authors declare that the research was conducted in the absence of any commercial or financial relationships that could be construed as a potential conflict of interest.

## Publisher's Note

All claims expressed in this article are solely those of the authors and do not necessarily represent those of their affiliated organizations, or those of the publisher, the editors and the reviewers. Any product that may be evaluated in this article, or claim that may be made by its manufacturer, is not guaranteed or endorsed by the publisher.
