# Supplementary material for: The Data Efficiency of Deep Learning Is Degraded by Unnecessary Input Dimensions
Source: Front Comput Neurosci. 2022 Jan 31;16:760085. doi: 10.3389/fncom.2022.760085 (PMC8842477; doi:10.3389/fncom.2022.760085)
Supplement: Supplementary file 1 [file Presentation_1.pdf]

APPENDIX 1 DATA GENERATION DETAILS

We provide further details about the data generation. In Appendix 1.1, we discuss the linearly separable dataset, in Appendix 1.2 we discuss the non-linearly separable dataset, and Appendix 1.3 divides in three parts, where we introduce the synthetic MNIST, the natural MNIST, and the Stanford dogs datasets.

Appendix 1.1 Linearly Separable Dataset

For the binary classification datasets, we generate the samples using a linear teacher network $\mathbf{y}_i = W^*\mathbf{x}_i$, with $W^* \in \mathbb{R}^{2\times 30}$ (*ie.,* $o = 2$, $p = 30$) components, generated from a standard Gaussian distribution $(\mu = 0, \sigma = 1)$. Each input sample dimension is also drawn from a standard Gaussian distribution. To generate the labels, we quantize the output to two categories. The *task-unrelated* dimensions have $\sigma = 0.1$, while *task-related* dimensions are linear combinations of the p (= 30) dimensions of the dataset, where the weights of the combinations are selected from a standard Gaussian distribution.

Appendix 1.2 Non-Linearly Separable Dataset

For the non-linearly separable binary classification datasets, we generate the samples by extracting those from a mixture of three Gaussians. Each Gaussian has 30 independent components each with its own mean value μ_c^g, where g denotes one Gaussian among the three, and c denotes the dimension. We assign a value to μ_c^g, by randomly extracting from a Gaussian distribution ($\mu = 0$, $\sigma = 0.5$).

Appendix 1.3 Object Recognition Datasets

Synthetic MNIST. In Figure 2A, we show the different experiments on the Synthetic MNIST datasets. In all of these, the MNIST digit is normalized to have values between 0 and 1. For the experiments with *task-unrelated* dimensions, the image size increases as we add pixels with random values at the edge, which constitute the *task-unrelated* dimensions. These random values are the absolute value of numbers extracted from a zero-mean Gaussian distribution with $\sigma = 0.2$. The MNIST digit edge has size 28 across all the *task-unrelated* versions for this dataset, while the image edge is equal to $[40, 56, 80, 120, 160, 188, 200]$. For the experiment with *task-related* dimensions, we upscale the MNIST digit using bi-cubic interpolation. After the upscale, the image size is equal to $[28, 36, 40, 56, 80, 120, 160]$. For the experiment with *task-related/unrelated* dimensions, we use the same aforementioned procedure to generate the *task-unrelated* and *task-related* dimensions. We fix the image size to 200×200 pixels, while the upscaled MNIST digit edge is equal to $[28, 36, 40, 56, 80, 120, 160]$. The amount of training examples per class for all the experiments is equivalent to $n_{tr} = [1, 2, 5, 10, 20, 50, 100, 300, 500, 1000]$.

Natural MNIST. We embed the MNIST digits on a natural background from the PLACES dataset (Zhou et al., 2014), as in (Volokitin et al., 2017). We generate the *task-related/unrelated* by sampling without replacement a normalized natural image of size 256×256 pixels. We superimpose at its center an upscaled and normalized digit from MNIST. The upscaled MNIST digit assumes sizes $[200, 150, 80, 28]$ as shown in Figure 2B. The number of examples per class corresponds to $n_{tr} = [20, 50, 100, 200, 300, 500, 1000]$.

Stanford Dogs. All the images from the Stanford Dogs dataset, for all the five cases considered in Figure 2C, are resized to 227×227 pixels. We subtract to the RGB channels the values $R_{\text{mean}} = 123.68$, $G_{\text{mean}} = 116.78$, and $B_{\text{mean}} = 103.94$, as done for recentering the ImageNet dataset. We split the original training set into a training and a validation set, the former consisting of 90 examples per class (breed), the latter consisting of 10 examples per class. The amount of training examples per class used to compute the AUTC is $n_{tr} = [23, 45, 90]$.

APPENDIX 2 ARCHITECTURE AND OPTIMIZATION DETAILS

In Appendix 2.1, we report the optimization details of the solution to the linear problem, using the pseudo-inverse. Architectures and optimization protocols of networks used on the linearly separable classes

Table 1. CNN for object recognition. From the top: number of filters *C or nodes *L at each hidden layer, filters size and max pooling sizes for *C; activation functions following *C or *L.

	iC	iiC	iiiC	iL	iiL
#filters/#nodes	32	64	64	64	10
filter, pooling	var, var	3, 2	3, none	none	none
act. function	ReLU	ReLU	None	ReLU	soft-max

and the MNIST datasets are respectively in Appendix 2.2 and Appendix 2.3. Appendix 2.4 contains the optimization procedure used on the Stanford Dogs dataset.

Appendix 2.1 Linear classifier (pseudo-inverse)

We repeat the classification experiments 10 times. We use the pseudo-inverse solution as described in (Appendix 3.1) below.

Appendix 2.2 MLP

Architectures. We consider MLPs with one hidden layer consisting of 128 nodes and a soft-max operation preceding the output.

Optimization Protocol. MLPs trained on the linearly separable binary classification task share the same optimization protocol. We fix the maximum number of epochs at 100. The convergence criterion is based on early stopping, with tolerance value 10^{-4} on the validation loss and patience of 8 epochs. Optimal batch size and learning rate are selected through a grid search procedure, respectively, over the arrays $[2, 10, 32, 50]$ and $[10^{-5}, 10^{-4}, 10^{-3}, 10^{-2}]$. We eliminate batch size values from the hyper-parameters search anytime those are equal to or larger than the number of training examples. We apply a reduction factor $1/10$ on the learning rate as the validation loss shows variations smaller than 10^{-4} over five epochs. The initialization of the weights adopted across networks and layers is the Glorot uniform, *ie.,* uniform distribution in the interval $[-u, u]$, where $u = \sqrt{6/(\text{fan}_{\text{in}} + \text{fan}_{\text{out}})}$, with fan_{in} and fan_{out} respectively number of input and output units. We report mean and standard deviation of the AUTC values across three repetitions of each experiment. The optimization protocol of MLP for MNIST is the same as the CNN detailed next.

Appendix 2.3 CNNs on synthetic MNIST and natural MNIST

Architectures.

The CNNs consist of three convolutional layers (iC-iiiC) followed by a flatten operation and two fully connected layers (iL and iiL). In Table 1 we report: at the first row the number of filters or nodes, depending on the layer; at the second row, the size of filters and max-pooling operations; and at the last row, the non-linearity used, if any. The filters and max-pool operations have square dimensions, and their sizes at the first layers vary across experiments.

In Table 2, we report filter and max-pooling sizes corresponding to different receptive fields at the first layer. The AUTC and test accuracy for these filter/max-pooling configurations are evaluated on natural MNIST, in Figure 2B.

Optimization Protocols. CNNs on synthetic MNIST share the same optimization protocol of MLPs. MLPs are provided with a vectorized version of the image. Models are optimized using stochastic gradient descent with null momentum. Batch size and learning rate are selected using a grid search procedure. The learning rate array has values $[1, 8 \cdot 10^{-1}, 5 \cdot 10^{-1}, 2 \cdot 10^{-1}, 10^{-1}, 10^{-2}, 10^{-3}, 10^{-4}, 10^{-5}, 5 \cdot 10^{-6}, 10^{-6}]$, the batch

Table 2. Filters and max-Pooling sizes at the first layer for CNNs trained on Natural MNIST.

	Adapt	$r = 9$	$r = 4.5$	$r = 2.3$	$r = 1$
filter	$r \cdot 3$	27	14	7	3
pooling	$r \cdot 2$	18	9	5	2

size array is $[10, 32, 50, 100]$. We eliminate batch size values from the hyper-parameters search anytime those are equal to or larger than the number of training examples. The convergence criterion is based on early stopping, convergence is reached when the validation loss shows across 10 repetitions variations smaller than 10^{-6}. The maximum amount of epochs is fixed at 500. We reduce the learning rate by a factor $1/10$ when variations of the validation loss are smaller than 10^{-4} across 5 epochs. The initialization of the weights values across architectures is based on the Glorot uniform distribution, or uniform distribution in the interval $[-u, u]$, where $u = \sqrt{6/(\text{fan}_{\text{in}} + \text{fan}_{\text{out}})}$, with fan_{in} and fan_{out} respectively number of input and output units.

The natural MNIST optimization protocol shares most of its hyper-parameters with the synthetic MNIST case. The main variation is a different grid of learning rates, fixed at $[5 \cdot 10^{-1}, 2 \cdot 10^{-1}, 10^{-1}, 10^{-2}, 10^{-3}, 10^{-4}]$.

We report mean and standard deviation of log-AUTC and test accuracy across two repetitions of the experiments of synthetic MNIST, and three repetitions on natural MNIST.

Appendix 2.4 ResNet-18 on Stanford Dogs

Optimization. Across all the experiments, the learning rate is equal to $1.28 \cdot 10^{-1}$ at the first iteration. We then divide it by a factor of 10 every time we reach a plateau of the validation accuracy. All the models are trained until we reach the plateau for the smallest learning rate considered, which is $1.28 \cdot 10^{-3}$. In all experiments, we observed no improvement of the validation accuracy after the plateau at learning rate $1.28 \cdot 10^{-2}$.

APPENDIX 3 THEORETICAL ANALYSIS OF LINEAR MODELS WITH UNNECESSARY INPUT DIMENSIONS

In this section, we analyze linear, shallow networks trained with the square loss. In Appendix 3.1, we introduce the theoretical analysis and in Appendix 3.2, we report experiments to illustrate the theoretical results. In this context, the additional input dimensions as defined in the following are not the most general and the results involving them rely on assumptions detailed below. Nonetheless, this theoretical sketch provides useful intuitions about the interplay between necessary and unnecessary input dimensions. For a more general and recent theoretical discussion see (Bernstein and Yue, 2021).

Appendix 3.1 Pseudo-inverse solution for linear, shallow networks

We use (X, Y) to denote a dataset where $X = [\mathbf{x}_1 \cdots \mathbf{x}_n] \in \mathbb{R}^{p \times n}$ contains n independent observations of p uncorrelated features, and $Y = [\mathbf{y}_1 \cdots \mathbf{y}_n] \in \mathbb{R}^{o \times n}$ denotes the respective ground-truth output. We define p as the minimal dimensionality. We aim at estimating the function f, such that, for every example $(\mathbf{x}_i, \mathbf{y}_i)$, $\mathbf{y}_i = f(\mathbf{x}_i)$ holds approximately. The choice of a linear network $\hat{f}(\mathbf{x}) = W\mathbf{x}$, with $W \in \mathbb{R}^{o \times p}$ and the square loss as cost function assures that, even for $p > n$ (overparameterized case), optimization through gradient descent leads to a unique solution that corresponds, among all, to the one with minimum norm, namely the pseudo-inverse (Schölkopf et al., 2001):

$$W^{+} = Y(X^{\top}X)^{-1}X^{\top}, \text{ with } p > n. \tag{1}$$

In the following, we introduce the theoretical solution to the linear model when the dataset contains *task-related*, *task-unrelated* and *task-related/unrelated* dimensions. We assume that the model operates in the overparameterized regime, $p > n$, which is the most common in practice.

Task-related dimensions. We assume that the unnecessary *task-related* dimensions come from a linear transformation $T \in \mathbb{R}^{d\times p}$ of the vector of p minimal dimensions. Thus, the dataset with unnecessary dimensions is the result of the transformation $F = [\mathbb{I}_p^\top \ T^\top]^\top \in \mathbb{R}^{(p+d)\times p}$, where $\mathbb{I}_p$ denotes the identity matrix of size p. The pseudo-inverse solution of the linear model in Eq. (1) becomes the following:

$$W^+ = Y(X^\top F^\top F X)^{-1} X^\top F^\top. \tag{2}$$

We further assume F to be a tight frame (Daubechies, 1992), *ie.,* there is a unique scaling factor $a > 0$ such that $\|F\mathbf{v}\|^2 = a\|\mathbf{v}\|^2$ holds for any vector $\mathbf{v} \in \mathbb{R}^p$. Then, Eq. (2) corresponds to $W^+ = a^{-1}Y(X^\top X)^{-1}X^\top F^\top$, which is the same as the linear model learned without *task-related* dimensions in Eq. (1) (the scaling constant a compensates with the term at the numerator at prediction). Thus, *task-related* dimensions do not affect the linear model if these are based on a tight frame, otherwise, *task-related* dimensions may change the solution of the linear model.

Task-unrelated dimensions. For each example i, we denote $\mathbf{n}_i \in \mathbb{R}^d$ a vector of d *task-unrelated* dimension independent from $\mathbf{x}_i \in \mathbb{R}^p$. Thus, the new input vector to the linear model is $[\mathbf{x}_i^\top \ \mathbf{n}_i^\top]^\top$. Intuitively, if $\mathbf{n}_i^\top$ is randomly generated, it is expected that as the number of *task-unrelated* dimensions increase there will be more overfitting. Yet, we will show several exceptions to this intuition. To illustrate the effect of these unnecessary input dimensions, we consider *task-unrelated* dimensions distributed as a Gaussian, with zero mean, diagonal covariance and same variance σ^2, *ie.,* $(\mathbf{n}_i)_\ell \sim \mathcal{N}(0, \sigma^2)$, $\ell = 1, \ldots, d, i = 1, \ldots, n$.

We assume that $p + d > n$ (*ie.,* overparameterization), and then, the pseudo-inverse solution of the linear model in Eq. (1) becomes the following:

$$W^+ = Y(X^\top X + N^\top N)^{-1}[X^\top \ N^\top]. \tag{3}$$

From this solution, we can compute the output of the linear model for a given test sample $[\mathbf{x}_{\text{ts}}^\top \ \mathbf{n}_{\text{ts}}^\top]^\top$ as in the following:

$$\begin{aligned} W^+[\mathbf{x}_{\text{ts}}^\top \ \mathbf{n}_{\text{ts}}^\top]^\top &= Y(X^\top X + N^\top N)^{-1}[X^\top \ N^\top][\mathbf{x}_{\text{ts}}^\top \ \mathbf{n}_{\text{ts}}^\top]^\top \\ &= Y(X^\top X + N^\top N)^{-1}(X^\top \mathbf{x}_{\text{ts}} + N^\top \mathbf{n}_{\text{ts}}) \\ &= Y(X^\top X + N^\top N)^{-1}X^\top \mathbf{x}_{\text{ts}} + Y(X^\top X + N^\top N)^{-1}N^\top \mathbf{n}_{\text{ts}}. \end{aligned} \tag{4}$$

Note that the first term contains the minimal dimensions and the second term the *task-unrelated* dimensions. In the following, we approximate this expression to a more interpretable one.

First, we develop the last product of the second term, *ie.,* $N^\top \mathbf{n}_{\text{ts}}$, which we write in a more explicit form:

$$N^\top \mathbf{n}_{\text{ts}} = \left[\mathbf{n}_1^\top \mathbf{n}_{\text{ts}} \ \cdots \ \mathbf{n}_n^\top \mathbf{n}_{\text{ts}}\right]^\top \tag{5}$$

$$= \left[\sum_{\ell=1}^{d} (\mathbf{n}_1)_\ell (\mathbf{n}_{\text{ts}})_\ell \ \cdots \ \sum_{\ell=1}^{d} (\mathbf{n}_n)_\ell (\mathbf{n}_{\text{ts}})_\ell\right]^\top. \tag{6}$$

Recall the assumption that all $(\mathbf{n}_i)_\ell$ and $(\mathbf{n}_{\text{ts}})_\ell$ are samples of two independent zero-mean Gaussian random variables with the same variance σ^2. For very large d values, we can approximate the average realization of a random variable through its expected value (law of large numbers). Thus, each sum in Eq. (6) approximates the expectation value of the product of two independent random variables, multiplied by a d factor. This can be written as the product of their expectation values, which can be neglected for zero-mean Gaussian variables for large values of d, *ie.,*

$$\sum_{\ell=1}^{d} (\mathbf{n}_i)_\ell (\mathbf{n}_{\text{ts}})_\ell \simeq 0. \tag{7}$$

In our experiments below, we find that with just few hundreds of unnecessary dimensions, this term can be approximated to be equal to 0. Therefore, for any test sample, we can derive from Eq. 6 the following expression:

$$N^\top \mathbf{n}_{\text{ts}} \simeq \mathbf{0} \tag{8}$$

Proceeding in a similarly, for the term $N^\top N$ in Eq. (4), related to *task-unrelated* dimensions, for $i \neq j$, we have:

$$\mathbf{n}_i^\top \mathbf{n}_j = \sum_{\ell=1}^{d} (\mathbf{n}_i)_\ell (\mathbf{n}_j)_\ell \simeq 0. \tag{9}$$

When $i = j$, the following approximation holds:

$$\mathbf{n}_i^\top \mathbf{n}_i = \sum_{\ell=1}^{d} (\mathbf{n}_i)_\ell^2 \simeq d\sigma^2. \tag{10}$$

This is because the expectation value coincides with the variance of the zero-mean Gaussian variable. Given Eqs. (9) and (10), we obtain the following approximation:

$$N^\top N \simeq d\sigma^2 \mathbb{I}_n. \tag{11}$$

Finally, the approximations in Eqs. (8) and (11) lead to the following approximation of the prediction of the linear network for large d:

$$W^+ [\mathbf{x}_{\text{ts}}^\top \, \mathbf{n}_{\text{ts}}^\top]^\top \simeq Y (X^\top X + d\sigma^2 \mathbb{I}_n)^{-1} X^\top \mathbf{x}_{\text{ts}}, \tag{12}$$

where $[\mathbf{x}_{\text{ts}}^\top \, \mathbf{n}_{\text{ts}}^\top]^\top$ is a test sample.

We can observe by analysing Eq. (12) that $d\sigma^2 \mathbb{I}_n$ corresponds to the Tikhonov regularization term, with regularization parameter $\lambda = d\sigma^2$. Thus, a large number of *task-unrelated* dimensions following a Gaussian distribution leads to a network trained on the dataset without *task-unrelated* dimensions, biased towards small ℓ_2 norm weights. This intrinsic regularization term usually will not have a positive effect in the test accuracy, as we do not have control to adjust the strength of the regularizer *ie.,* $d\sigma^2$ is given by the dataset. It may lead to underfitting with respect to the minimal dimensions of the dataset, or equivalently, to overfitting to the dataset with the unnecessary dimensions. Yet, this regularizer may have positive effects in some

situations. In Appendix 3.2, we introduce a regression problem with corrupted labels, *ie.,* $y_i = f(x_i) + \varepsilon_i$, that can benefit from the regularization effect of *task-unrelated* dimensions.

Combining task-related and task-unrelated dimensions. We now assume that a percentage $\nu \in [0, 1]$ of unnecessary input dimensions are *task-related* and the rest are *task-unrelated*. We borrow the results introduced above for each of these two types of unnecessary dimensions, and combine them.

Let $N = [\mathbf{n}_1 \cdots \mathbf{n}_n] \in \mathbb{R}^{d(1-\nu)\times n}$ be the matrix of *task-unrelated* dimensions, and $TX = [T\mathbf{x}_i \cdots T\mathbf{x}_n] \in \mathbb{R}^{d\nu\times n}$ be the *task-related* dimensions, in which $T \in \mathbb{R}^{d\nu\times p}$ is a generic linear transformation. Thus, the input samples of the training set in matrix form is equal to $[X^\top \; N^\top \; (TX)^\top]^\top$, which is equivalent to the concatenation of these terms.

We can express the input matrix using the frame formalism as

$$\begin{pmatrix} X \\ N \\ TX \end{pmatrix} = F \begin{pmatrix} X \\ N \end{pmatrix}, \tag{13}$$

$$\text{where } F = \begin{pmatrix} \mathbb{I}_p & \mathbf{0}_{p\times d(1-\nu)} \\ \mathbf{0}_{d(1-\nu)\times p} & \mathbb{I}_{d(1-\nu)} \\ T & \mathbf{0}_{d\nu\times d(1-\nu)} \end{pmatrix}, \tag{14}$$

and $\mathbf{0}$ denotes the matrix with null entries and dimensions as specified in the subscripts. Recall that Eq. (2) introduced the solution of a linear model when the input is multiplied by a frame. Thus, we develop Eq. (2) in order to obtain a more interpretable approximation that provides insights about the effect of *task-related* and *unrelated* dimensions.

We first evaluate the term $F^\top F$ from Eq. (2) with the frame in Eq. 14, which yields the following:

$$F^\top F = \left(\begin{array}{c|c} \mathbb{I}_p + T^\top T & \mathbf{0}_{p\times d(1-\nu)} \\ \hline \mathbf{0}_{d(1-\nu)\times p} & \mathbb{I}_{d(1-\nu)} \end{array}\right), \tag{15}$$

and it is invertible. By substituting this term in the pseudo-inverse solution of Eq. (2), we obtain

$$\begin{aligned} W^+ &= Y \left(\left[X^\top \; N^\top \right] F^\top F \begin{bmatrix} X \\ N \end{bmatrix} \right)^{-1} \left[X^\top N^\top \right] F^\top \\ &= Y \left(X^\top \left(\mathbb{I}_p + T^\top T \right) X + N^\top N \right)^{-1} \left[X^\top \left[\mathbb{I}_p \; T^\top \right] \; N^\top \right]. \end{aligned} \tag{16}$$

This result does not rely on any assumption on the distribution of the *task-unrelated* dimensions, neither on a specific form of the linear transformation T. To get a clearer picture of the effect of unnecessary input dimensions, in the following we make four assumptions: (i) the *task-related* dimensions are generated by repeating k times the minimal set of dimensions, such that $k = (\nu d)/p$; (ii) the vector components for the *task-unrelated* dimensions are drawn from zero-mean Gaussian distributions sharing the same variance value; (iii) $d(1-\nu)$ number of *task-unrelated* dimensions is large; (iv) the correlation between necessary input dimensions at training and test is very large (there are no cases in which the necessary dimensions for two samples are orthogonal).

Given assumption (i), T is a tight frame and the quantity $T^\top T$ corresponds to the k times identity in $\mathbb{R}^p$. Also, the term $\mathbb{I}_p + T^\top T$ is equivalent to $(k+1)\mathbb{I}_p$ because the minimal set of dimensions are repeated k times.

The solution in Eq. (16) applied on a new test sample $\left[\mathbf{x}_{\text{ts}}^\top\, \mathbf{n}_{\text{ts}}^\top\, (T\mathbf{x}_{\text{ts}})^\top\right]^\top$, becomes to the following expression:

$$W^+ \left[\mathbf{x}_{\text{ts}}^\top\, \mathbf{n}_{\text{ts}}^\top\, (T\mathbf{x}_{\text{ts}})^\top\right]^\top = \\ Y\left((k+1)X^\top X + N^\top N\right)^{-1}\left((k+1)X^\top \mathbf{x}_{\text{ts}} + N^\top \mathbf{n}_{\text{ts}}\right). \quad (17)$$

Using assumptions (ii) and (iii), we leverage on the law of large numbers as in the *task-unrelated* case introduced before. Here, the sum over the *task-unrelated* components consists of $d(1-\nu)$ terms, and the following two approximations holds: $N^\top \mathbf{n}_{\text{ts}} \simeq \mathbf{0}$ and $N^\top N \simeq d(1-\nu)\sigma^2\mathbb{I}_n$. Thus, the prediction for a test sample corresponds to

$$W^+ \left[\mathbf{x}_{\text{ts}}^\top\, \mathbf{n}_{\text{ts}}^\top\, (T\mathbf{x}_{\text{ts}})^\top\right]^\top \simeq \\ Y\left((k+1)X^\top X + d(1-\nu)\sigma^2\mathbb{I}_n\right)^{-1} X^\top \mathbf{x}_{\text{ts}}(k+1) = \\ Y\left(X^\top X + \frac{pd(1-\nu)}{d\nu+p}\sigma^2\mathbb{I}_n\right)^{-1} X^\top \mathbf{x}_{\text{ts}}. \quad (18)$$

Observe that this expression is again the pseudo-inverse solution with Tikhonov regularization as for the *task-unrelated* dimensions in Eq. (12), except that here regularization parameter is equal to $\frac{pd(1-\nu)}{d\nu+p}\sigma^2$. The regularizer term depends on the ratio between the number of *task-unrelated* and *task-related* dimensions, ν. Note that as the number of *task-related* dimensions increases, in the limit of ν tending to 1, the problem is equivalent to the one formulated for the minimal dimensions, with null contribution from the unnecessary input dimensions as the regularizer parameter would tend to 0. Thus, the number of *task-related* dimensions help to adjust the regularization effect of the *task-unrelated* dimensions, and hence, it can alleviate its negative effects.

Appendix 3.2 Regression with corrupted output

We now analyze a problem in practice that follows up from the theoretical analysis in the previous section, and that serves to show that the regularization effect of the unnecessary input dimensions can be helpful in some cases.

We generate an overparameterized regression dataset, with $p = 10$ minimal dimensions, $o = 4$ output dimensions, and $d = 500$ unnecessary *task-unrelated* dimensions. The p minimal input dimensions are drawn from $\mathcal{N}(0, \sigma^2_{\text{input}})$, zero-mean Gaussian distribution with a shared variance value. The number of

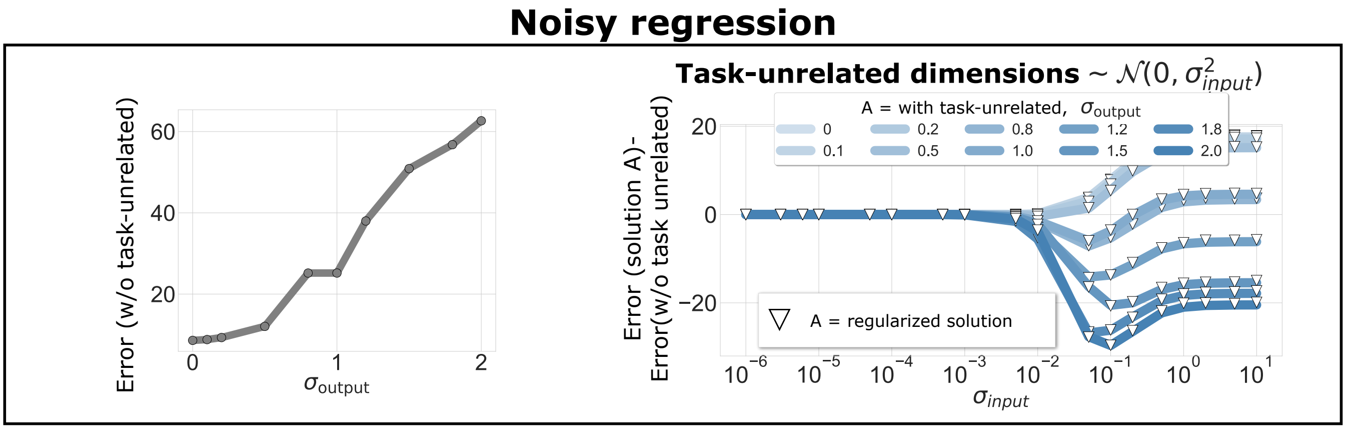

Figure 3. Regression with corrupted output. Left: prediction error when there are no unnecessary dimensions, as function of the output corruption. Right: analysis of prediction error as function of the variance of the *task-unrelated* dimensions/ regularization (with regularization value $\lambda = d\sigma^2_{input}$). On the y-axis, difference between test errors of either the solution "*with task-unrelated*" or regularized, and "*without task-unrelated* solution".

training examples across experiments is $n = 7$. The corrupted output for sample i is

$$\mathbf{y}_i = W^*\mathbf{x}_i + \varepsilon, \tag{19}$$

with zero-mean Gaussian distributed random variable $\varepsilon \sim \mathcal{N}(0, \sigma^2_{\text{output}})$.

We compare three different baselines. We refer to the "*with task-unrelated* solution" as the solution computed from the samples with *task-unrelated* input dimensions, *ie.,* $\left[\mathbf{x}_i^\top \; \mathbf{n}_i^\top\right]^\top$, using Eq. (12). When there is no *task-unrelated* dimensions, we refer to the "*without task-unrelated* solution", as in Eq. (1). Finally, we also compute the *Tikhonov* regularized solution with $\lambda = d\sigma^2_{\text{input}}$ regularization parameter, on the dataset without unnecessary input dimensions. The prediction errors for the three baselines correspond respectively to the following:

$$\text{Error}(\textit{with task-unrelated}) =$$

$$\left\langle \left\| Y(X^\top X + N^\top N)^{-1}[X^\top \; N^\top][\mathbf{x}_{\text{ts}}^\top \; \mathbf{n}_{\text{ts}}^\top]^\top - \mathbf{y}_{\text{ts}} \right\|_2^2 \right\rangle, \tag{20}$$

$$\text{Error}(\textit{w/o task-unrelated}) =$$

$$\left\langle \left\| Y(X^\top X)^{-1} X^\top \mathbf{x}_{\text{ts}} - \mathbf{y}_{\text{ts}} \right\|_2^2 \right\rangle, \tag{21}$$

$$\text{Error}(\textit{Tikhonov}) =$$

$$\left\langle \left\| Y(X^\top X + \lambda \mathbb{I}_n)^{-1} X^\top \mathbf{x}_{\text{ts}} - \mathbf{y}_{\text{ts}} \right\|_2^2 \right\rangle, \tag{22}$$

where the sample average $\langle \cdot \rangle$ is computed on the test examples.

In Fig. 3 on the left, we report the test error computed as in Eq. 21. As expected, as the corruption in the ground-truth output increases, we observe consistently higher test errors. On the right of Fig. 3, we quantify

the difference between Eq. (20) and Eq.(21), as the variance of *task-unrelated* dimensions increases. Each blue curve is related to a different level of output corruption. For small values of σ_{input}, *task-unrelated* dimensions do not have any effect on the test error. As σ_{input} increases, *task-unrelated* dimensions harm the solution for very small output corruption, while gradually benefiting the prediction as the output corruption increases due to its regularization effect.

The white triangles on the left of Fig. 3 correspond to the difference between Eq. (20) and Eq. (22). These points follow each of the blue curves, showing that the assumptions and approximations we made are reasonable.

Overall, these results depict the effect of *task-unrelated* dimensions, which can effectively help to improve the test accuracy when the ground-truth output is corrupted.

APPENDIX 4 SUPPLEMENTAL RESULTS

Appendix 4.1 Supplementary Results on synthetic MNIST

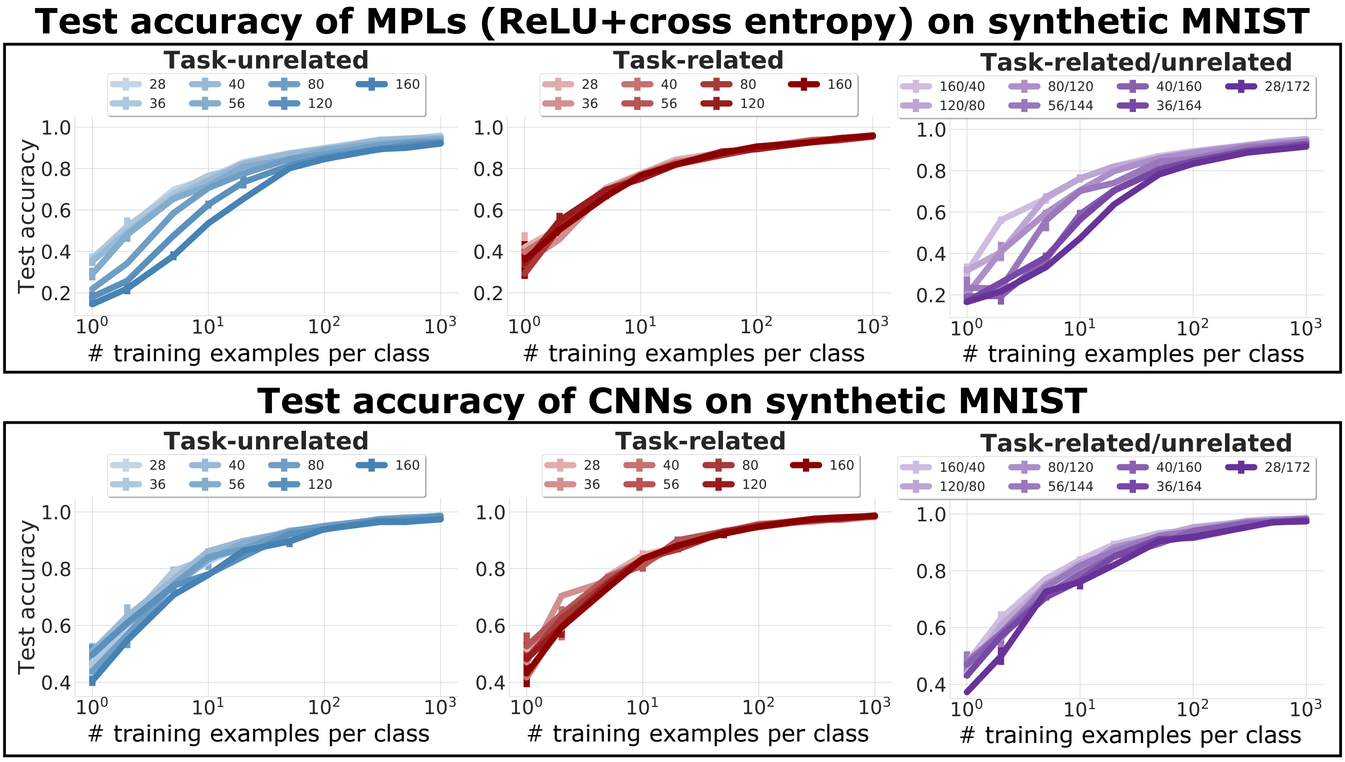

Figure 4. Test accuracy for different number of training examples for the MLP with ReLU trained with cross entropy loss (top row) and CNNs (bottom row) across the three scenarios on synthetic MNIST.

In Figure 4, we report the test accuracy of the MLP and CNN, trained on synthetic MNIST, for the three types of unnecessary dimensions and the different amount of dimensions. These curves are used to compute the log-AUTC in Figure 2A.

Appendix 4.2 Supplemental Results on natural MNIST

On the top left of Figure 5, we report the CNN's test accuracy obtained with different filters sizes and max-pooling sizes when the amount of unnecessary dimensions, as the amount of training examples per class ($n_{tr} = 1000$) are the largest we tested. All the versions of the CNN achieve almost perfect

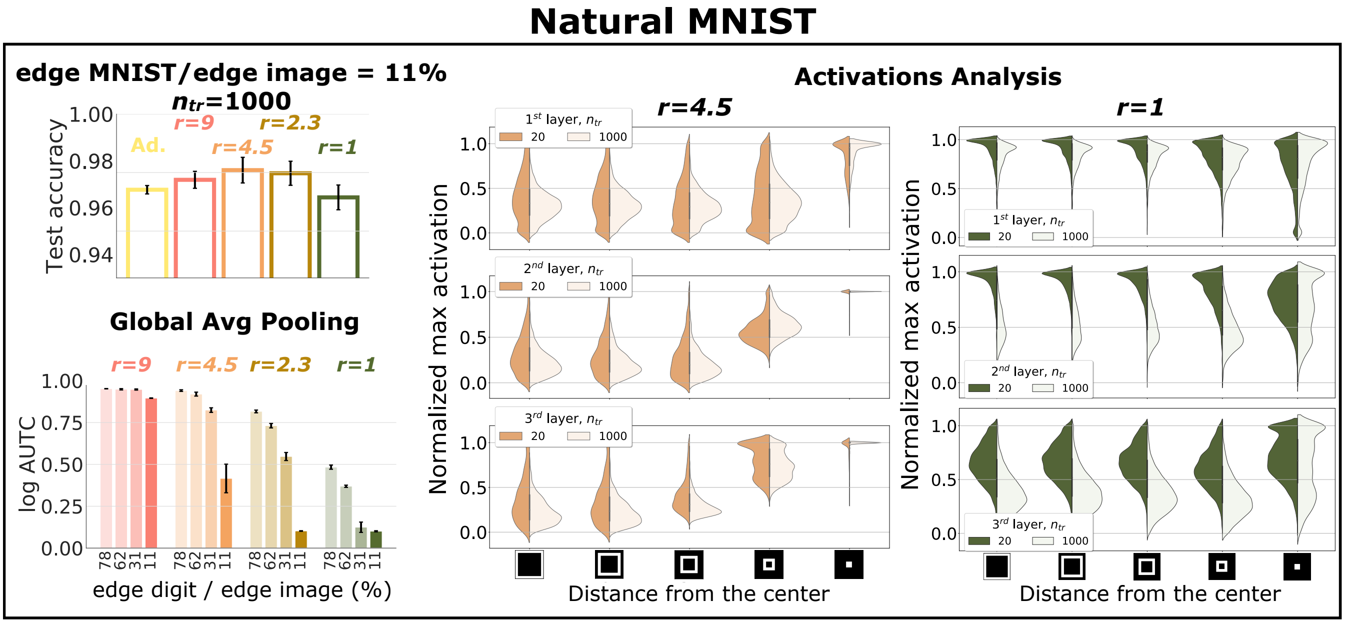

Figure 5. Top left: Test accuracy for the highest amount of *task-unrelated* dimensions, with the largest amount of training examples. Bottom left: log AUTC with architectures with global average pooling. On the right, normalized maximum activations for the two networks $r = 4.5$ and $r = 1$ in Figure 2B.

generalization, emphasizing that data efficiency's gaps due to unnecessary input dimensions are hard to observe when abundant amounts of examples are available.

On the bottom left of Figure 5, we show the log AUTC values for networks trained on Natural MNIST with a global average pooling before the fully connected layers. The global average pooling yields fully connected layers that have the same amount of trainable parameters independently of the receptive field sizes. Results show that the CNN with the largest receptive fields size, $r = 9$, leads to the highest data efficiency among all the different r we tested. CNNs with the smallest receptive field size, $r = 1$, heavily suffer from the unnecessary input dimensions. Despite the reduction of free parameters caused by the global pooling, depending on the receptive field size, the data efficiency of the network could dramatically drop due to *task-unrelated* input dimensions.

Neural Mechanisms to Discard Object's Background in CNNs. We investigate the networks' mechanisms that facilitate discarding the unnecessary dimensions. To do so, we provide evidence that supports the hypothesis that efficient CNNs have kernels tuned to discard the object's background.

To compute the activations of a network, we extract at each layer the neural activity of 1000 test samples after the non-linearity and the max-pooling (wherever the max-pooling transformation applies). For each image and at every layer, we compute the maximum activation across spatial dimensions and filters, which we use to normalize the representation of the image at that layer. We grouped the representation values at each layer in five sets, depending on their distance from the center (regions). We store the maximum normalized value for each region.

In Figure 5, we report the distribution of the maximum normalized values for the five regions (left to right corresponds to the furthest to the closest region to the center). We report results for the most (in orange) and least (in green) data efficient networks without global average pooling (respectively $r = 4.5$ and $r = 1$). Also, we compare the activations of the two networks when trained on the smallest $n_{tr} = 20$ (dark) and largest $n_{tr} = 1000$ (light) sets, for edge digit / edge image $= 11\%$.

When the network is trained with more examples (light violin plots), the neurons respond more to the object compared to the background. Yet, when the networks are trained with few examples (dark violin plots), the differences in terms of neural activity are minor between the object and its background. Note that this phenomenon is much more pronounced for the most data efficient architecture ($r = 4.5$). These results suggest that the data efficiency of the network is driven by the emergence of kernels that can detect the object while not responding to the background.
